# Therapeutic Effects of Natural Compounds and Small Molecule Inhibitors Targeting Endoplasmic Reticulum Stress in Alzheimer’s Disease

**DOI:** 10.3389/fcell.2021.745011

**Published:** 2021-09-01

**Authors:** Xun Gao, Yuanyuan Xu

**Affiliations:** Key Laboratory of Zoonosis Research, Ministry of Education, College of Animal Science, Jilin University, Changchun, China

**Keywords:** Alzheimer’s disease, apoptosis, endoplasmic reticulum stress, neuroprotection, unfolded protein response

## Abstract

Alzheimer’s disease (AD) is the most common neurodegenerative disease, characterized by progressive cognitive impairment and memory loss. So far, the pathogenesis of AD has not been fully understood. Research have shown that endoplasmic reticulum (ER) stress and unfolded protein response (UPR) participate in the occurrence and development of AD. Furthermore, various studies, both *in vivo* and *in vitro*, have shown that targeting ER stress and ER stress-mediated apoptosis contribute to the recovery of AD. Thus, targeting ER stress and ER stress-mediated apoptosis may be effective for treating AD. In this review, the molecular mechanism of ER stress and ER stress-mediated apoptosis, as well as the therapeutic effects of some natural compounds and small molecule inhibitors targeting ER stress and ER stress-mediated apoptosis in AD will be introduced.

## Introduction

The endoplasmic reticulum (ER) is a crucial organelle of eukaryotic cells, whose functions include protein synthesis and folding, lipid biogenesis and calcium metabolism (Schwarz and Blower, 2016). Numerous cellular stresses, such as disequilibrium of calcium homeostasis, redox imbalance, changes in protein glycosylation, or protein folding defects in the ER, can trigger ER stress response, causing accumulation of unfolded or misfolded proteins in the ER lumen (Senft and Ronai, 2015). In order to maintain the homeostasis of the ER, the unfolded protein response (UPR), an integrated signal transduction pathway, is activated (Walter and Ron, 2011). UPR can inhibit protein transcription and translation, promote the degradation of misfolded proteins and increase the ability of correct protein folding (Hetz, 2012). However, under chronic or excessive ER stress, UPR fails to maintain the homeostasis of ER, then the apoptosis signaling pathway is activated, leading to a variety of diseases, including Alzheimer’s disease (AD) (Uddin et al., 2020).

AD is the most common neurodegenerative disease, characterized by progressive cognitive impairment and memory loss. Besides, abnormal accumulation of misfolded proteins in the brain, such as β-amyloid (Aβ) peptide and hyperphosphorylated tau protein, is the main neuropathological hallmark of AD (Villain and Dubois, 2019; Ghemrawi and Khair, 2020). So far, the pathogenesis of AD has not been fully understood, and there is no effective treatment for this disease. The existing treatments can only relieve the symptoms or slow down the progression of the disease, but the patients can’t be cured (Morris et al., 2018). Therefore, it is necessary to find a novel and effective treatment for AD.

Recently, research have shown that ER stress and UPR participate in the occurrence and development of AD (Hoozemans et al., 2009; Salminen et al., 2009; Uddin et al., 2020). Furthermore, various studies, both *in vivo* and *in vitro*, have shown that targeting ER stress and ER stress-mediated apoptosis contribute to the recovery of AD (Xu et al., 2018; Zhu et al., 2019; Song et al., 2020; Oliveira et al., 2021). Thus, targeting ER stress and ER stress-mediated apoptosis may be effective for treating AD. In this review, the molecular mechanism of ER stress and ER stress-mediated apoptosis, as well as the therapeutic effects of some natural compounds and small molecule inhibitors targeting ER stress and ER stress-mediated apoptosis in AD will be introduced.

## ER Stress, UPR, and Apoptosis

### ER Stress-Mediated UPR Signaling Pathways

**FIGURE 1:**
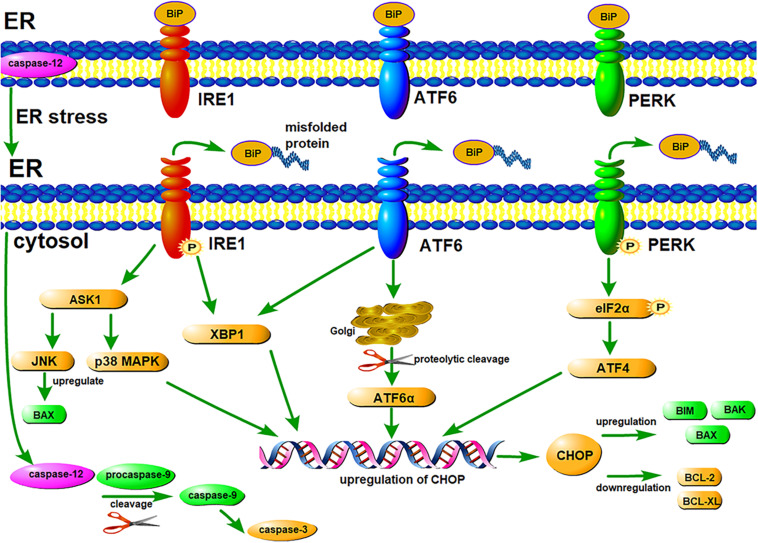
ER stress-mediated UPR signaling pathways and apoptosis signaling pathways. Three ER transmembrane proteins, IRE1, ATF6, and PERK, are involved in UPR signaling pathways. Under normal conditions, these proteins are combined with ER chaperone BiP, and located in the ER membrane in an inactive state. Under ER stress, when a misfolded protein binds to BiP, BiP dissociates from IRE1, ATF6, and PERK. Subsequently, PERK and IRE1 undergo autophosphorylation, and ATF6 undergoes proteolytic cleavage. Downstream signaling pathways of UPR are initiated, thereby maintaining ER homeostasis. However, under chronic or excessive ER stress, UPR fails to maintain the homeostasis of ER, then apoptosis signaling pathways are activated. All the three ER transmembrane proteins can up-regulate CHOP, leading to apoptosis by regulating the expression of some BCL-2 family members. Activated IRE1 can activate JNK, inducing apoptosis by up-regulating the expression of some pro-apoptotic proteins. Caspase-12 can induce apoptosis by activating the caspase cascade reaction.

Early ER stress will induce UPR, which might be protective to cells (Walter and Ron, 2011). Three ER transmembrane proteins are involved in UPR signaling pathways: PERK (protein kinase RNA-like ER kinase), IRE1 (inositol-requiring enzyme 1) and ATF6 (activating transcription factor 6) (Chen and Brandizzi, 2013; Hillary and FitzGerald, 2018; Rozpedek et al., 2019). Under physiological conditions, these proteins are combined with ER chaperone BiP (immunoglobulin binding protein, also called glucose-regulated protein 78, GRP78), and located in the ER membrane in an inactive state (Carrara et al., 2015; Ghemrawi and Khair, 2020). However, under ER stress, when a misfolded or unfolded protein binds to BiP, BiP is dissociated from these transmembrane proteins, then downstream signaling pathways of UPR are initiated (Figure 1; Ron and Walter, 2007; Parmar and Schroder, 2012). These three transmembrane proteins play a key role in the three signaling pathways of UPR, promoting the correct folding of unfolded and misfolded proteins, thus maintaining ER homeostasis.

PERK is a type I ER transmembrane protein with a serine/threonine protein kinase domain (Rozpedek et al., 2019). Under mild ER stress, PERK first undergoes autophosphorylation, and then eukaryotic initiation factor 2α (eIF2α) is activated by phosphorylation. Phosphorylated eIF2α can block protein synthesis and reduce the protein load of ER (Cnop et al., 2017).

IRE1 is also a type I ER transmembrane protein with endonuclease activities, which can be divided into two isforms, IRE1α and IRE1β (Chen and Brandizzi, 2013). When ER stress occurs, IRE1α is first activated by autophosphorylation and dimerization (Hetz et al., 2011). Then, through unconventional splicing of X-box binding protein 1 (XBP1) mRNA, an active transcription factor, XBP1s, is produced. XBP1s can promote the correct folding of unfolded and misfolded porteins, thereby restore ER homeostasis (Hollien et al., 2009).

ATF6 is a type II transmembrane protein, which encodes a basic leucine zipper (bZIP) transcription factor (Hillary and FitzGerald, 2018). In conditions of ER stress, ATF6 moves from the ER to the Golgi, where it undergoes proteolytic cleavage. Subsequently, the active transcription factor, ATF6α, is produced and transferred to the nucleus, where it regulates the expression of UPR related genes, such as CHOP, BiP, and XBP1 (Wu et al., 2007; Ghemrawi and Khair, 2020).

### Chronic or Excessive ER Stress-Mediated Apoptosis Signaling Pathways

On the contrary, under chronic or excessive ER stress, UPR fails to maintain the homeostasis of ER, then the apoptosis signaling pathways are activated, even resulting in cell death (Senft and Ronai, 2015; Iurlaro and Munoz-Pinedo, 2016). ER stress can induce apoptosis through three major signaling pathways: CHOP pathway, JNK pathway and caspase-12 pathway (Figure 1; Hu et al., 2018).

#### PERK-CHOP Pathway

Phosphorylated eIF2α allows the translation of activating transcription factor 4 (ATF4), an activator of apoptosis-related genes (Hu et al., 2018). Subsequently, AFT4 up-regulates the expression of several apoptosis-related genes, for example, C/EBP homologous protein (CHOP) (Oyadomari and Mori, 2004). CHOP can regulate the expression of some BCL-2 family members, including the down-regulation of anti-apoptotic proteins like BCL-2 and BCL-XL, and the up-regulation of pro-apoptotic proteins like BIM, BAK, and BAX (Hata et al., 2015). Furthermore, the oligomerization of BAK-BAX will lead to the release of cytochrome c, which causes apoptosis through the mitochondrial pathway (Brenner and Mak, 2009).

#### IRE1-JNK Pathway

Activated IRE1 can activate apoptotic-signaling kinase-1 (ASK1) (Ron and Hubbard, 2008). Then downstream kinases, such as Jun-N-terminal kinase (JNK) and p38 mitogen-activated protein kinase (p38 MAPK), are activated (Urano et al., 2000). JNK can up-regulate the expression of pro-apoptotic proteins, such as BIM and PUMA, leading to the activation of BAX and subsequent apoptosis (Deng et al., 2001). Meanwhile, p38 MAPK can promote the transcription of CHOP, as well as regulate the expression of some BCL-2 family members (Oyadomari and Mori, 2004; Hata et al., 2015).

#### Caspase-12 Pathway

ER stress can activate caspase-12, and then the caspase cascade is activated (Iurlaro and Munoz-Pinedo, 2016). Activated caspase-12 moves from the ER to the cytosol, where it cleaves procaspase-9 to form caspase-9 (Szegezdi et al., 2003). Caspase-9 in turn activates caspase-3, the main executioner of apoptosis (Lossi et al., 2018).

## Applications of Natural Compounds and Small Molecule Inhibitors Targeting ER Stress and ER Stress-Mediated Apoptosis in Treating Alzheimer’s Disease

Researches have shown that ER stress and UPR participate in the occurrence and development of AD (Hoozemans et al., 2009; Salminen et al., 2009; Uddin et al., 2020). During AD, the continuous accumulation of Aβ or p-tau leads to unbalanced ER calcium homeostasis, abnormal protein folding and ER stress (Sobow et al., 2004; Mondragon-Rodriguez et al., 2012). Neuronal cells are especially sensitive to protein misfolding, therefore, the excessive accumulation of Aβ or p-tau will result in synaptic dysfunction and apoptosis, even neuronal death (Remondelli and Renna, 2017; Ghemrawi and Khair, 2020). In addition, some UPR-related proteins, such as phosphorylated PERK, eIF2α and IRE1 were found in AD brains (Devi and Ohno, 2014; Duran-Aniotz et al., 2017; Uddin et al., 2020). Meanwhile, ER stress markers, including GRP78, CHOP and caspase-12, are up-regulated in AD patients (Santos and Ferreira, 2018).

Recently, various studies, both *in vivo* and *in vitro*, have shown that targeting ER stress and ER stress-mediated apoptosis contribute to the recovery of AD (Xu et al., 2018; Zhu et al., 2019; Song et al., 2020; Oliveira et al., 2021). Several active components extracted from the plants and small molecule inhibitors of UPR signaling pathways and other signaling pathways have displayed good therapeutic effects on AD models. A brief summary of some natural compounds and small molecule inhibitors targeting ER stress in AD studies is shown in Table 1.

**TABLE 1:** Natural compounds and small molecule inhibitors targeting ER stress and ER stress-mediated apoptosis in treating Alzheimer’s disease.

**Agents**	**AD models**	**Mechanisms**	**References**
**Natural compounds**			
Schisandrin	Streptozotocin- induced rat model	GRP78↓ CHOP↓ caspase-12↓	Song et al., 2020
Resveratrol and crocin	Aβ_2__5__–__3__5_ -induced rat model	GRP78↓ CHOP↓ caspase-3↓ BAX↓ BCL-2↑	Lin et al., 2019
Gas	Tg2576 mouse model	eIF2α↓	Zhang et al., 2016
Quercetin	APP23 mouse model	p-eIF2α↓ ATF4↓	Hayakawa et al., 2015
Bajijiasu	Aβ_2__5__–__3__5_-induced PC12 cell model; APP/PS1 mouse model	IRE1α↓ PERK↓ eIFα↓ CHOP↓	Chen et al., 2013; Xu et al., 2018
Ginsenoside Rg1	APP/PS1 rat model	GRP78↓ IRE1↓ TRAF2↓ p-JNK↓ caspase-3↓	Mu et al., 2015
GE and its pure compounds	Aβ-induced BV2 cell model	IRE1α↓ XBP1↓ PERK↓ p-eIF2α↓ CHOP↓ caspase-3↓	Lee et al., 2012
Taurine	H_2_O_2_-induced PC12 cell model; hypoxia- and glutamate-induced primary neuronal cell model;	Cleaved ATF6↓ p-IRE1↓ GRP78↓ CHOP↓ BIM↓ BCL-2↑	Pan et al., 2010, 2012
	APP/PS1 mouse model; Aβ-infusion mouse model	Unknown	Kim et al., 2014; Jang et al., 2017
SAC	Aβ-induced cell models (PC12 cells; hippocampal neurons; rat organotypic hippocampal slice cultures)	Caspase-12↓	Ito et al., 2003; Kosuge et al., 2003; Imai et al., 2007
**Small molecule inhibitors**			
LDN-0060609	Thapsigargin-induced DI TNC1 cell model	p-eIF2α↓	Rozpedek et al., 2019
ISRIB	APP_*SWE*_/PS1_△__*E*__9_ mouse model	ATF4↓	Oliveira et al., 2021
Xestospongin C	APP/PS1 mouse model	GRP78↓ CHOP↓ caspase-12↓	Wang et al., 2019
DA-CH3	APP_*SWE*_/PS1_△__*E*__9_ mouse model	Akt↑	Panagaki et al., 2018
Bpv	APP/PS1 mouse model	GRP78↓ CHOP↓ BAX↓ p-AKT/AKT↑	Cui et al., 2017
ATAN	Aβ-induced cell model (rat organotypic hippocampal slice cultures)	Caspase-12↓	Imai et al., 2007

*BAX, BCL-2-associated X protein; BCL-2, B-cell lymphoma 2; BiP, immunoglobulin heavy chain-binding protein; CHOP, C/EBP homologous protein; eIF2, eukaryotic initiation factor 2; GRP78, glucose-regulated Protein 78; PERK, protein kinase RNA-like ER kinase; IRE1, inositol-requiring protein 1; JNK, Jun-N-terminal kinase; p38 MAPK, p38 mitogen-activated protein kinase; TRAF2, tumor necrosis factor receptor-associated 2.*

### Targeting PERK-CHOP Signaling Pathway

The PERK-CHOP signaling pathway plays an essential role in inducing cell apoptosis. In the early stage of ER stress, activated PERK can further activate eIF2α, which can block protein synthesis and exert a protective effect on cells (Rozpedek et al., 2019). However, under chronic or excessive ER stress, PERK-CHOP signaling pathway will be activated, leading to apoptosis (Hu et al., 2018).

#### Schisandrin (Sch)

Schisandrin (Sch) is an active component extracted from schisandra chinensis Baill (Zhu et al., 2019). In the Streptozotocin (STZ)-induced AD rats, after the treatment of Sch, the expression of ER stress markers, including GRP78, CHOP, and cleaved caspase-12, was obviously decreased (Song et al., 2020). Moreover, Sch also improved the learning and memory capacity of STZ-induced AD rats, by enhancing the activity of Sirtuin 1 (SIRT1, an enzyme contributes to the acquisition and maintenance of memory) (Song et al., 2020).

#### Resveratrol and Crocin

In the Aβ_2__5–3__5_-induced AD rats, both enhanced expression of GRP78, CHOP, caspase-3, BAX and attenuated expression of BCL-2 were observed in the hippocampal CA1 region (Hippo) and prefrontal cortical (PFC), while the situation was reversed by treatment with resveratrol (a polyphenolic stilbenoid, present in grapes, mulberries, peanuts and other plants; Malaguarnera, 2019) and crocin (a non-tetraquinone pigment extracted from *Crocus sativus* L.; Shafahi et al., 2018). Furthermore, improved learning and memory ability and decreased number of apoptotic neurons were also detected in AD rats (Lin et al., 2019).

#### Gastrodin (Gas)

Tg2576 transgenic mouse is an AD model with high expression of β-site APP-cleaving enzyme 1 (BACE1), which is a rate-limiting enzyme for Aβ generation and could aggravate the process of AD (O’Connor et al., 2008). Gastrodin (Gas), an active component of Gastrodia elata Blume, was found to improve learning and memory abilities and attenuate intracellular oxidative stress of Tg2576 mice (Zhang et al., 2016). Moreover, Gas down-regulated BACE1 expression via inhibiting activation of PKR and eIF2α (Zhang et al., 2016).

#### Quercetin

In the APP23 AD model mice, after long-term feeding with quercetin, a polyhydroxylated flavonoid, the expression of growth arrest and DNA damaged-inducible gene 34 (GADD34) was induced, leading to the down-regulation of phosphorylated-eIF2α and ATF4 (Ohta et al., 2011; Hayakawa et al., 2015). This resulted in improvement of memory in aged AD mice, and delayed deterioration in memory of the mice at the early stage of AD (Hayakawa et al., 2015).

#### LDN-0060609

Compound LDN-0060609 is a small molecule PERK inhibitor (Rozpedek et al., 2019). In rat astrocytic DI TNC1 cell line, LDN-0060609 pretreatment attenuated the pro-apoptotic, PERK-dependent signaling pathway induced by thapsigargin (Th) treatment. It significantly inhibited eIF2α phosphorylation and increased cell survival *in vitro*. Notably, LDN-0060609 has no cytotoxicity to DI TNC1 cells and also has no influence on cell cycle progression (Rozpedek et al., 2019; Rozpedek-Kaminska et al., 2020). Therefore, it may contribute to prevention against apoptosis and neurodegeneration in AD without the cytotoxic effect.

#### ISRIB

ISRIB is a small molecule integrated stress response (ISR) inhibitor, which can reverse the phosphorylation of eIF2α and inhibit the downstream targets of eIF2α, such as ATF4, CHOP, and GADD34 (Sidrauski et al., 2015). In an AD mouse model induced by intracerebroventricular injection of amyloid-β oligomers (AβOs), ISRIB treatment counteracted the increase in ATF4 protein level, protecting mice from long-term memory impairment (Oliveira et al., 2021). Furthermore, ISRIB was shown to attenuate translational repression, restore synaptic plasticity and memory in transgenic APP_*SWE*_/PS1_△__*E*__9_ AD mice (Oliveira et al., 2021).

#### Xestospongin C (XeC)

Xestospongin C (XeC), a compound isolated from the Xestospongia species, is a reversible IP_3_ receptor antagonist (Gafni et al., 1997). It was found that XeC could improve the cognitive behavior of APP/PS1 AD mice. XeC also reduced the number of Aβ plaques and down-regulated the expression of GRP78, CHOP and caspase-12 in APP/PS1 mice (Wang et al., 2019). In addition, XeC significantly ameliorated Aβ_1–4__2_-induced neuronal apoptosis and intracellular Ca^2+^ overload in primary cultured hippocampal neurons (Wang et al., 2019).

### Targeting IRE1-JNK Signaling Pathway

Activated IRE1 can activate JNK, leading to the up-regulation of pro-apoptotic proteins and subsequent apoptosis (Urano et al., 2000; Deng et al., 2001). Activation of IRE1-JNK signaling pathway plays a key role in ER stress-mediated apoptosis.

#### Bajijiasu

Bajijiasu (also known as bajisu) is a natural active ingredient isolated from Morinda officinalis (Chen et al., 2014). It was shown to play a protective role against Aβ_25__–__35_-induced neurotoxicity in PC12 cells (Chen et al., 2013). In a double transgenic APP/PS1 mouse model of AD, oral administration of bajijiasu improved learning and memory abilities of APP/PS1 mice. Also, Bajijiasu protected neurons from apoptosis by down-regulating the expression of IRE1α, PERK, eIF2α, and CHOP. Moreover, reduced ROS and MDA levels in both the hippocampus and cortex were detected (Xu et al., 2018).

#### Ginsenoside Rg1

Ginsenoside Rg1 is a steroidal saponin highly abundant in ginseng (Wu et al., 2013). In a APP/PS1 rat model, AD rats were fed with 0.5% Rg1-enriched food to investigate the neuroprotective effects of Rg1. After Rg1 treatment, the accumulation of Aβ plaque and neurofbrillary tangles (NFTs) was significantly decreased in the AD rats, as well as the reduced expression of caspase-3 and the number of apoptotic cells. Furthermore, down-regulation of GRP78, IRE1, and p-JNK was observed in the AD rats, indicating that Rg1 exhibited neuroprotective effects by inhibiting the ER stress-mediated JNK apoptotic pathway (Mu et al., 2015).

#### Gastrodia Elata (GE) and Its Pure Compounds

In the BV2 mouse microglial cells, Aβ not only induced cytotoxicity and apoptosis but also promoted the expression of IRE1α, XBP1, PERK, phosphorylated eIF2α, CHOP, caspase-3 (Lee et al., 2012). However, after treatment with Gastrodia elata (GE) and its pure compounds, gastrodin (Gas) and 4-hydroxybenzyl alcohol (4HBA), these ER stress-relevant proteins were down-regulated. Moreover, Gas and 4HBA inhibited the neurotoxicity induced by Aβ and increased cells viability (Lee et al., 2012).

### Targeting ATF6-AKT Signaling Pathway

Under ER stress, the ATF6 pathway can mediate cell survival by regulating the activation of Akt and the expression of GRP78 and CHOP (Cui et al., 2017).

#### Taurine

Taurine, a free amino acid, is abundant in the brain and plays an important role in the central nervous system (Seidel et al., 2019). It was found that taurine can protect PC12 cells against H_2_O_2_-induced ER stress, leading to the increase in cell viability, as well as the down-regulation of GRP78, CHOP, and BIM (Pan et al., 2010). Meanwhile, in primary neuronal cultures, taurine can also modulate hypoxia- and glutamate-induced ER stress by down-regulating the expression of caspase-12, CHOP, cleaved ATF6 and p-IRE1 (Pan et al., 2012). Later, in APP/PS1 transgenic AD mouse model, oral administration of taurine significantly ameliorated cognitive deficits of the adult AD mice (Kim et al., 2014). Further study conducted in the oligomeric Aβ-infusion AD mouse model showed that taurine can ameliorate cognitive impairment by directly binding to oligomeric Aβ (Jang et al., 2017). Nevertheless, mechanisms underlying taurine mediated cognitive improvement still need further elucidation.

#### Dipotassium Bisperoxo-(5-Hydroxypyridine-2-Carboxyl)-Oxovanadate (bpv)

Phosphatase and tensin homolog deleted on chromosome ten (PTEN) is a tumor suppressor, which plays an essential role in regulating neuronal survival or apoptosis (Kitagishi and Matsuda, 2013). Dipotassium bisperoxo-(5-hydroxypyridine-2-carboxyl)-oxovanadate (bpv), a PTEN inhibitor, could decrease apoptosis and suppress the expression of ER stress related protein GRP78, CHOP, and Bax in APP/PS1 transgenic AD mice. Further study showed that the neuroprotective role of bpv in APP/PS1 mice was mediated by activation of PI3K/AKT signaling pathways (Cui et al., 2017).

#### DA-CH3

Chronic treatment with DA-CH3, a novel dual GLP-1/GIP receptor agonist (Holscher, 2018), could rescue the spatial acquisition and memory impairments of APP_*SWE*_/PS1_△__*E*__9_ mice. Additionally, excessive plaque deposition, gliosis and synaptic damage was ameliorated in the APP_*SWE*_/PS1_△__*E*__9_ brain. Further research found that the alleviated ER stress and autophagy impairments in the APP_*SWE*_/PS1_△__*E*__9_ mouse brain might be attributed to the up-regulation of the Akt activation (Panagaki et al., 2018).

### Targeting Caspase-12 Signaling Pathway

ER stress can induce mitochondrial apoptosis by regulating Bcl-2 family members, such as Bcl-2 and Bcl-XL. However, persistent ER stress will activate caspase-12, and further activate caspase-3, finally triggering cell apoptosis (Iurlaro and Munoz-Pinedo, 2016).

S-allyl-L-cysteine (SAC) is an organosulfur compound extracted from aged garlic (Moriguchi et al., 1996). It was shown that SAC could prevent neuronal death induced by Aβ in PC12 cells (Ito et al., 2003), cultured hippocampal neurons (Kosuge et al., 2003), and rat organotypic hippocampal slice cultures (OHCs) (Imai et al., 2007). Besides, Aβ-induced increase in caspase-12 protein expression was suppressed by SAC in cultured hippocampal neurons and OHCs. Furthermore, SAC also decreased the Aβ-induced intracellular reactive oxygen species (ROS) levels in hippocampal neurons (Kosuge et al., 2003). In addition, Z-ATAN-fmk (ATAN), a specific caspase-12 inhibitor, could markedly suppress neurotoxicity induced by Aβ in OHCs (Imai et al., 2007).

## Discussion

It is proved that ER stress and UPR play an essential role in the occurrence and development of AD (Devi and Ohno, 2014; Duran-Aniotz et al., 2017; Uddin et al., 2020). The related findings, both *in vivo* and *in vitro*, have shown that many natural compounds and small molecular inhibitors targeting ER stress and ER stress-mediated neuronal apoptosis contribute to the recovery of AD (Xu et al., 2018; Zhu et al., 2019; Song et al., 2020; Oliveira et al., 2021). However, there are still a lot of questions to be considered if we want to convert these agents into a safe and reliable drug for clinical application, such as the safety, efficacy, and applicability.

Nevertheless, due to the species differences between humans and mice, the existing AD mouse models cannot fully simulate the pathologic and clinical features of human AD, which limits their application in preclinical studies of AD (de Bem et al., 2020). Therefore, it is necessary to develop animal models more similar to human AD to test the safety and efficacy of these agents (Medina and Avila, 2014). Recently, with the development of efficient genome editing technology, larger animals, such as rabbits, pigs and non-human primates, can be used to construct models of AD (Bosze et al., 2003; Chan, 2013; Yang and Wu, 2018). By using the CRISPR/Cas9 system and base editing system, these animal models can accurately simulate the gene mutation sites of human AD, and provide an ideal platform for AD pathogenesis research and preclinical evaluation (Tu et al., 2015; Gaudelli et al., 2017). In the near future, more unknown mechanisms between ER stress and AD will be clarified, which will offer more safe and effective therapeutic strategies to AD.

## Author Contributions

XG and YX initiated the project, wrote, revised, and finalized the manuscript. XG searched the database. Both authors contributed to the article and approved the submitted version.

## Conflict of Interest

The authors declare that the research was conducted in the absence of any commercial or financial relationships that could be construed as a potential conflict of interest.

## Publisher’s Note

All claims expressed in this article are solely those of the authors and do not necessarily represent those of their affiliated organizations, or those of the publisher, the editors and the reviewers. Any product that may be evaluated in this article, or claim that may be made by its manufacturer, is not guaranteed or endorsed by the publisher.

## References

[B1] Bosze Z. Hiripi L. Carnwath J. W. Niemann H. (2003). The transgenic rabbit as model for human diseases and as a source of biologically active recombinant proteins. *Transgenic Res.* 12 541–553. 14601653 10.1023/a:1025816809372

[B2] Brenner D. Mak T. W. (2009). Mitochondrial cell death effectors. *Curr. Opin. Cell Biol.* 21 871–877. 10.1016/j.ceb.2009.09.004 19822411

[B3] Carrara M. Prischi F. Nowak P. R. Kopp M. C. Ali M. M. (2015). Noncanonical binding of BiP ATPase domain to Ire1 and Perk is dissociated by unfolded protein CH1 to initiate ER stress signaling. *Elife* 4:e03522. 10.7554/eLife.03522 PMC4337721 25692299

[B4] Chan A. W. (2013). Progress and prospects for genetic modification of nonhuman primate models in biomedical research. *ILAR J.* 54 211–223. 10.1093/ilar/ilt035 24174443 PMC3814401

[B5] Chen D. L. Zhang P. Lin L. Shuai O. Zhang H. M. Liu S. H. (2013). Protective effect of Bajijiasu against beta-amyloid-induced neurotoxicity in PC12 cells. *Cell. Mol. Neurobiol.* 33 837–850. 10.1007/s10571-013-9950-7 23812758 PMC11497914

[B6] Chen D. L. Zhang P. Lin L. Zhang H. M. Deng S. D. Wu Z. Q. (2014). Protective effects of bajijiasu in a rat model of Abeta(2)(5)(-)(3)(5)-induced neurotoxicity. *J. Ethnopharmacol.* 154 206–217. 10.1016/j.jep.2014.04.004 24742752

[B7] Chen Y. Brandizzi F. (2013). IRE1: ER stress sensor and cell fate executor. *Trends Cell Biol.* 23 547–555. 23880584 10.1016/j.tcb.2013.06.005 PMC3818365

[B8] Cnop M. Toivonen S. Igoillo-Esteve M. Salpea P. (2017). Endoplasmic reticulum stress and eIF2alpha phosphorylation: the Achilles heel of pancreatic beta cells. *Mol. Metab.* 6 1024–1039. 10.1016/j.molmet.2017.06.001 28951826 PMC5605732

[B9] Cui W. Wang S. Wang Z. Wang Z. Sun C. Zhang Y. (2017). Inhibition of PTEN Attenuates Endoplasmic Reticulum Stress and Apoptosis via Activation of PI3K/AKT Pathway in Alzheimer’s Disease. *Neurochem. Res.* 42 3052–3060. 10.1007/s11064-017-2338-1 28819903

[B10] de Bem A. F. Krolow R. Farias H. R. De Rezende V. L. Gelain D. P. Moreira J. C. F. (2020). Animal Models of Metabolic Disorders in the Study of Neurodegenerative Diseases: an Overview. *Front. Neurosci.* 14:604150. 10.3389/fnins.2020.604150 33536868 PMC7848140

[B11] Deng X. Xiao L. Lang W. Gao F. Ruvolo P. May W. S. Jr. (2001). Novel role for JNK as a stress-activated Bcl2 kinase. *J. Biol. Chem.* 276 23681–23688. 10.1074/jbc.m100279200 11323415

[B12] Devi L. Ohno M. (2014). PERK mediates eIF2alpha phosphorylation responsible for BACE1 elevation, CREB dysfunction and neurodegeneration in a mouse model of Alzheimer’s disease. *Neurobiol. Aging* 35 2272–2281. 10.1016/j.neurobiolaging.2014.04.031 24889041 PMC4127890

[B13] Duran-Aniotz C. Cornejo V. H. Espinoza S. Ardiles A. O. Medinas D. B. Salazar C. (2017). IRE1 signaling exacerbates Alzheimer’s disease pathogenesis. *Acta Neuropathol.* 134 489–506. 10.1007/s00401-017-1694-x 28341998

[B14] Gafni J. Munsch J. A. Lam T. H. Catlin M. C. Costa L. G. Molinski T. F. (1997). Xestospongins: potent membrane permeable blockers of the inositol 1,4,5-trisphosphate receptor. *Neuron* 19 723–733. 10.1016/s0896-6273(00)80384-0 9331361

[B15] Gaudelli N. M. Komor A. C. Rees H. A. Packer M. S. Badran A. H. Bryson D. I. (2017). Programmable base editing of AT to GC in genomic DNA without DNA cleavage. *Nature* 551 464–471. 10.1038/nature24644 29160308 PMC5726555

[B16] Ghemrawi R. Khair M. (2020). Endoplasmic Reticulum Stress and Unfolded Protein Response in Neurodegenerative Diseases. *Int. J. Mol. Sci.* 21:6127. 10.3390/ijms21176127 32854418 PMC7503386

[B17] Hata A. N. Engelman J. A. Faber A. C. (2015). The BCL2 Family: key Mediators of the Apoptotic Response to Targeted Anticancer Therapeutics. *Cancer Discov.* 5 475–487. 10.1158/2159-8290.cd-15-0011 25895919 PMC4727530

[B18] Hayakawa M. Itoh M. Ohta K. Li S. Ueda M. Wang M. X. (2015). Quercetin reduces eIF2alpha phosphorylation by GADD34 induction. *Neurobiol. Aging* 36 2509–2518. 10.1016/j.neurobiolaging.2015.05.006 26070242

[B19] Hetz C. (2012). The unfolded protein response: controlling cell fate decisions under ER stress and beyond. *Nat. Rev. Mol. Cell Biol.* 13 89–102. 10.1038/nrm3270 22251901

[B20] Hetz C. Martinon F. Rodriguez D. Glimcher L. H. (2011). The unfolded protein response: integrating stress signals through the stress sensor IRE1alpha. *Physiol. Rev.* 91 1219–1243. 10.1152/physrev.00001.2011 22013210

[B21] Hillary R. F. FitzGerald U. (2018). A lifetime of stress: ATF6 in development and homeostasis. *J. Biomed. Sci.* 25:48. 10.1186/s12929-018-0453-1 PMC5968583 29801500

[B22] Hollien J. Lin J. H. Li H. Stevens N. Walter P. Weissman J. S. (2009). Regulated Ire1-dependent decay of messenger RNAs in mammalian cells. *J. Cell Biol.* 186 323–331. 10.1083/jcb.200903014 19651891 PMC2728407

[B23] Holscher C. (2018). Novel dual GLP-1/GIP receptor agonists show neuroprotective effects in Alzheimer’s and Parkinson’s disease models. *Neuropharmacology* 136 251–259. 10.1016/j.neuropharm.2018.01.040 29402504

[B24] Hoozemans J. J. Van Haastert E. S. Nijholt D. A. Rozemuller A. J. Eikelenboom P. Scheper W. (2009). The unfolded protein response is activated in pretangle neurons in Alzheimer’s disease hippocampus. *Am. J. Pathol.* 174 1241–1251. 10.2353/ajpath.2009.080814 19264902 PMC2671357

[B25] Hu H. Tian M. Ding C. Yu S. (2018). The C/EBP Homologous Protein (CHOP) Transcription Factor Functions in Endoplasmic Reticulum Stress-Induced Apoptosis and Microbial Infection. *Front. Immunol.* 9:3083. 10.3389/fimmu.2018.03083 30662442 PMC6328441

[B26] Imai T. Kosuge Y. Ishige K. Ito Y. (2007). Amyloid beta-protein potentiates tunicamycin-induced neuronal death in organotypic hippocampal slice cultures. *Neuroscience* 147 639–651. 10.1016/j.neuroscience.2007.04.057 17560726

[B27] Ito Y. Kosuge Y. Sakikubo T. Horie K. Ishikawa N. Obokata N. (2003). Protective effect of S-allyl-L-cysteine, a garlic compound, on amyloid beta-protein-induced cell death in nerve growth factor-differentiated PC12 cells. *Neurosci. Res.* 46 119–125. 10.1016/s0168-0102(03)00037-3 12725918

[B28] Iurlaro R. Munoz-Pinedo C. (2016). Cell death induced by endoplasmic reticulum stress. *FEBS J.* 283 2640–2652. 26587781 10.1111/febs.13598

[B29] Jang H. Lee S. Choi S. L. Kim H. Y. Baek S. Kim Y. (2017). Taurine Directly Binds to Oligomeric Amyloid-beta and Recovers Cognitive Deficits in Alzheimer Model Mice. *Adv. Exp. Med. Biol.* 975 233–241. 10.1007/978-94-024-1079-2_21 28849459

[B30] Kim H. Y. Kim H. V. Yoon J. H. Kang B. R. Cho S. M. Lee S. (2014). Taurine in drinking water recovers learning and memory in the adult APP/PS1 mouse model of Alzheimer’s disease. *Sci. Rep.* 4:7467. 10.1038/srep07467 PMC4264000 25502280

[B31] Kitagishi Y. Matsuda S. (2013). Diets involved in PPAR and PI3K/AKT/PTEN pathway may contribute to neuroprotection in a traumatic brain injury. *Alzheimers Res. Ther.* 5:42. 10.1186/alzrt208 24074163 PMC3978568

[B32] Kosuge Y. Koen Y. Ishige K. Minami K. Urasawa H. Saito H. (2003). S-allyl-L-cysteine selectively protects cultured rat hippocampal neurons from amyloid beta-protein- and tunicamycin-induced neuronal death. *Neuroscience* 122 885–895. 10.1016/j.neuroscience.2003.08.026 14643758

[B33] Lee G. H. Kim H. R. Han S. Y. Bhandary B. Kim D. S. Kim M. G. (2012). Gastrodia elata Blume and its pure compounds protect BV-2 microglial-derived cell lines against beta-amyloid: the involvement of GRP78 and CHOP. *Biol. Res.* 45 403–410. 10.4067/s0716-97602012000400013 23558999

[B34] Lin L. Liu G. Yang L. (2019). Crocin Improves Cognitive Behavior in Rats with Alzheimer’s Disease by Regulating Endoplasmic Reticulum Stress and Apoptosis. *Biomed. Res. Int.* 2019:9454913. 10.1155/2019/9454913 PMC6732583 31534969

[B35] Lossi L. Castagna C. Merighi A. (2018). Caspase-3 Mediated Cell Death in the Normal Development of the Mammalian Cerebellum. *Int. J. Mol. Sci.* 19:3999. 10.3390/ijms19123999 30545052 PMC6321612

[B36] Malaguarnera L. (2019). Influence of Resveratrol on the Immune Response. *Nutrients* 11:946. 10.3390/nu11050946 31035454 PMC6566902

[B37] Medina M. Avila J. (2014). The need for better AD animal models. *Front. Pharmacol.* 5:227. 10.3389/fphar.2014.00227 25386142 PMC4208405

[B38] Mondragon-Rodriguez S. Perry G. Zhu X. Boehm J. (2012). Amyloid Beta and tau proteins as therapeutic targets for Alzheimer’s disease treatment: rethinking the current strategy. *Int. J. Alzheimers Dis.* 2012:630182. 10.1155/2012/630182 PMC3310047 22482074

[B39] Moriguchi T. Saito H. Nishiyama N. (1996). Aged garlic extract prolongs longevity and improves spatial memory deficit in senescence-accelerated mouse. *Biol. Pharm. Bull.* 19 305–307. 10.1248/bpb.19.305 8850329

[B40] Morris G. Puri B. K. Walder K. Berk M. Stubbs B. Maes M. (2018). The Endoplasmic Reticulum Stress Response in Neuroprogressive Diseases: emerging Pathophysiological Role and Translational Implications. *Mol. Neurobiol.* 55 8765–8787. 10.1007/s12035-018-1028-6 29594942 PMC6208857

[B41] Mu J. S. Lin H. Ye J. X. Lin M. Cui X. P. (2015). Rg1 exhibits neuroprotective effects by inhibiting the endoplasmic reticulum stress-mediated c-Jun N-terminal protein kinase apoptotic pathway in a rat model of Alzheimer’s disease. *Mol. Med. Rep.* 12 3862–3868. 10.3892/mmr.2015.3853 26016457

[B42] O’Connor T. Sadleir K. R. Maus E. Velliquette R. A. Zhao J. Cole S. L. (2008). Phosphorylation of the translation initiation factor eIF2alpha increases BACE1 levels and promotes amyloidogenesis. *Neuron* 60 988–1009. 10.1016/j.neuron.2008.10.047 19109907 PMC2667382

[B43] Ohta K. Mizuno A. Li S. Itoh M. Ueda M. Ohta E. (2011). Endoplasmic reticulum stress enhances gamma-secretase activity. *Biochem. Biophys. Res. Commun.* 416 362–366. 10.1016/j.bbrc.2011.11.042 22115781

[B44] Oliveira M. M. Lourenco M. V. Longo F. Kasica N. P. Yang W. Ureta G. (2021). Correction of eIF2-dependent defects in brain protein synthesis, synaptic plasticity, and memory in mouse models of Alzheimer’s disease. *Sci. Signal* 14:eabc5429. 10.1126/scisignal.abc5429 33531382 PMC8317334

[B45] Oyadomari S. Mori M. (2004). Roles of CHOP/GADD153 in endoplasmic reticulum stress. *Cell Death Differ.* 11 381–389. 10.1038/sj.cdd.4401373 14685163

[B46] Pan C. Giraldo G. S. Prentice H. Wu J. Y. (2010). Taurine protection of PC12 cells against endoplasmic reticulum stress induced by oxidative stress. *J. Biomed. Sci.* 17:S17. 10.1186/1423-0127-17-S1-S17 PMC2994405 20804591

[B47] Pan C. Prentice H. Price A. L. Wu J. Y. (2012). Beneficial effect of taurine on hypoxia- and glutamate-induced endoplasmic reticulum stress pathways in primary neuronal culture. *Amino Acids* 43 845–855. 10.1007/s00726-011-1141-6 22080215

[B48] Panagaki T. Gengler S. Holscher C. (2018). The Novel DA-CH3 Dual Incretin Restores Endoplasmic Reticulum Stress and Autophagy Impairments to Attenuate Alzheimer-Like Pathology and Cognitive Decrements in the APPSWE/PS1DeltaE9 Mouse Model. *J. Alzheimers Dis.* 66 195–218. 10.3233/jad-180584 30282365

[B49] Parmar V. M. Schroder M. (2012). Sensing endoplasmic reticulum stress. *Adv. Exp. Med. Biol.* 738 153–168. 22399379 10.1007/978-1-4614-1680-7_10

[B50] Remondelli P. Renna M. (2017). The Endoplasmic Reticulum Unfolded Protein Response in Neurodegenerative Disorders and Its Potential Therapeutic Significance. *Front. Mol. Neurosci.* 10:187. 10.3389/fnmol.2017.00187 28670265 PMC5472670

[B51] Ron D. Hubbard S. R. (2008). How IRE1 reacts to ER stress. *Cell* 132 24–26. 10.1016/j.cell.2007.12.017 18191217

[B52] Ron D. Walter P. (2007). Signal integration in the endoplasmic reticulum unfolded protein response. *Nat. Rev. Mol. Cell Biol.* 8 519–529. 10.1038/nrm2199 17565364

[B53] Rozpedek W. Pytel D. Poplawski T. Walczak A. Gradzik K. Wawrzynkiewicz A. (2019). Inhibition of the PERK-Dependent Unfolded Protein Response Signaling Pathway Involved in the Pathogenesis of Alzheimer’s Disease. *Curr. Alzheimer Res.* 16 209–218. 10.2174/1567205016666190228121157 30819079

[B54] Rozpedek-Kaminska W. Siwecka N. Wawrzynkiewicz A. Wojtczak R. Pytel D. Diehl J. A. (2020). The PERK-Dependent Molecular Mechanisms as a Novel Therapeutic Target for Neurodegenerative Diseases. *Int. J. Mol. Sci.* 21:2108. 10.3390/ijms21062108 32204380 PMC7139310

[B55] Salminen A. Kauppinen A. Suuronen T. Kaarniranta K. Ojala J. (2009). ER stress in Alzheimer’s disease: a novel neuronal trigger for inflammation and Alzheimer’s pathology. *J. Neuroinflammation* 6:41. 10.1186/1742-2094-6-41 20035627 PMC2806266

[B56] Santos L. E. Ferreira S. T. (2018). Crosstalk between endoplasmic reticulum stress and brain inflammation in Alzheimer’s disease. *Neuropharmacology* 136 350–360. 10.1016/j.neuropharm.2017.11.016 29129774

[B57] Schwarz D. S. Blower M. D. (2016). The endoplasmic reticulum: structure, function and response to cellular signaling. *Cell Mol. Life Sci.* 73 79–94. 10.1007/s00018-015-2052-6 26433683 PMC4700099

[B58] Seidel U. Huebbe P. Rimbach G. (2019). Taurine: a Regulator of Cellular Redox Homeostasis and Skeletal Muscle Function. *Mol. Nutr. Food Res.* 63:e1800569. 10.1002/mnfr.201800569 30211983

[B59] Senft D. Ronai Z. A. (2015). UPR, autophagy, and mitochondria crosstalk underlies the ER stress response. *Trends Biochem. Sci.* 40 141–148. 10.1016/j.tibs.2015.01.002 25656104 PMC4340752

[B60] Shafahi M. Vaezi G. Shajiee H. Sharafi S. Khaksari M. (2018). Crocin Inhibits Apoptosis and Astrogliosis of Hippocampus Neurons Against Methamphetamine Neurotoxicity via Antioxidant and Anti-inflammatory Mechanisms. *Neurochem. Res.* 43 2252–2259. 10.1007/s11064-018-2644-2 30259275

[B61] Sidrauski C. Tsai J. C. Kampmann M. Hearn B. R. Vedantham P. Jaishankar P. (2015). Pharmacological dimerization and activation of the exchange factor eIF2B antagonizes the integrated stress response. *Elife* 4:e07314. 10.7554/eLife.07314 PMC4426669 25875391

[B62] Sobow T. Flirski M. Liberski P. P. (2004). Amyloid-beta and tau proteins as biochemical markers of Alzheimer’s disease. *Acta Neurobiol. Exp. (Wars)* 64 53–70. 15190680 10.55782/ane-2004-1491

[B63] Song L. Piao Z. Yao L. Zhang L. Lu Y. (2020). Schisandrin ameliorates cognitive deficits, endoplasmic reticulum stress and neuroinflammation in streptozotocin (STZ)-induced Alzheimer’s disease rats. *Exp. Anim.* 69 363–373. 10.1538/expanim.19-0146 32336744 PMC7445059

[B64] Szegezdi E. Fitzgerald U. Samali A. (2003). Caspase-12 and ER-stress-mediated apoptosis: the story so far. *Ann. N. Y. Acad. Sci.* 1010 186–194. 10.1196/annals.1299.032 15033718

[B65] Tu Z. Yang W. Yan S. Guo X. Li X. J. (2015). CRISPR/Cas9: a powerful genetic engineering tool for establishing large animal models of neurodegenerative diseases. *Mol. Neurodegener.* 10:35. 10.1186/s13024-015-0031-x PMC4524001 26238861

[B66] Uddin M. S. Tewari D. Sharma G. Kabir M. T. Barreto G. E. Bin-Jumah M. N. (2020). Molecular Mechanisms of ER Stress and UPR in the Pathogenesis of Alzheimer’s Disease. *Mol. Neurobiol.* 57 2902–2919. 10.1007/s12035-020-01929-y 32430843

[B67] Urano F. Wang X. Bertolotti A. Zhang Y. Chung P. Harding H. P. (2000). Coupling of stress in the ER to activation of JNK protein kinases by transmembrane protein kinase IRE1. *Science* 287 664–666. 10.1126/science.287.5453.664 10650002

[B68] Villain N. Dubois B. (2019). Alzheimer’s Disease Including Focal Presentations. *Semin. Neurol.* 39 213–226. 10.1055/s-0039-1681041 30925614

[B69] Walter P. Ron D. (2011). The unfolded protein response: from stress pathway to homeostatic regulation. *Science* 334 1081–1086. 10.1126/science.1209038 22116877

[B70] Wang Z. J. Zhao F. Wang C. F. Zhang X. M. Xiao Y. Zhou F. (2019). Xestospongin C, a Reversible IP3 Receptor Antagonist, Alleviates the Cognitive and Pathological Impairments in APP/PS1 Mice of Alzheimer’s Disease. *J. Alzheimers Dis.* 72 1217–1231. 10.3233/jad-190796 31683484

[B71] Wu J. Pan Z. Cheng M. Shen Y. Yu H. Wang Q. (2013). Ginsenoside Rg1 facilitates neural differentiation of mouse embryonic stem cells via GR-dependent signaling pathway. *Neurochem. Int.* 62 92–102. 10.1016/j.neuint.2012.09.016 23063465

[B72] Wu J. Rutkowski D. T. Dubois M. Swathirajan J. Saunders T. Wang J. (2007). ATF6alpha optimizes long-term endoplasmic reticulum function to protect cells from chronic stress. *Dev. Cell* 13 351–364. 10.1016/j.devcel.2007.07.005 17765679

[B73] Xu T. T. Zhang Y. He J. Y. Luo D. Luo Y. Wang Y. J. (2018). Bajijiasu Ameliorates beta-Amyloid-Triggered Endoplasmic Reticulum Stress and Related Pathologies in an Alzheimer’s Disease Model. *Cell Physiol. Biochem.* 46 107–117. 10.1159/000488414 29587274

[B74] Yang H. Wu Z. (2018). Genome Editing of Pigs for Agriculture and Biomedicine. *Front. Genet.* 9:360. 10.3389/fgene.2018.00360 30233645 PMC6131568

[B75] Zhang J. S. Zhou S. F. Wang Q. Guo J. N. Liang H. M. Deng J. B. (2016). Gastrodin suppresses BACE1 expression under oxidative stress condition via inhibition of the PKR/eIF2alpha pathway in Alzheimer’s disease. *Neuroscience* 325 1–9. 10.1016/j.neuroscience.2016.03.024 26987953

[B76] Zhu P. Li J. Fu X. Yu Z. (2019). Schisandra fruits for the management of drug-induced liver injury in China: a review. *Phytomedicine* 59:152760. 10.1016/j.phymed.2018.11.020 31004881

